# Neuroimaging of Sleep Disturbances in Movement Disorders

**DOI:** 10.3389/fneur.2018.00767

**Published:** 2018-09-11

**Authors:** Tayyabah Yousaf, Gennaro Pagano, Heather Wilson, Marios Politis

**Affiliations:** Neurodegeneration Imaging Group, Department of Basic and Clinical Neuroscience, Institute of Psychiatry, Psychology and Neuroscience, King's College London, London, United Kingdom

**Keywords:** neuroimaging, magnetic resonance imaging, positron emission tomography, sleep, Parkinson's disease, atypical Parkinsonism, REM behavior sleep disorder, excessive daytime sleepiness

## Abstract

Sleep dysfunction is recognized as a distinct clinical manifestation in movement disorders, often reported early on in the disease course. Excessive daytime sleepiness, rapid eye movement sleep behavior disorder and restless leg syndrome, amidst several others, are common sleep disturbances that often result in significant morbidity. In this article, we review the spectrum of sleep abnormalities across atypical Parkinsonian disorders including multiple system atrophy (MSA), progressive supranuclear palsy (PSP) and corticobasal syndrome (CBS), as well as Parkinson's disease (PD) and Huntington's disease (HD). We also explore the current concepts on the neurobiological underpinnings of sleep disorders, including the role of dopaminergic and non-dopaminergic pathways, by evaluating the molecular, structural and functional neuroimaging evidence based on several novel techniques including magnetic resonance imaging (MRI), functional magnetic resonance imaging (fMRI), diffusion tensor imaging (DTI), single-photon emission computed tomography (SPECT) and positron emission tomography (PET). Based on the current state of research, we suggest that neuroimaging is an invaluable tool for assessing structural and functional correlates of sleep disturbances, harboring the ability to shed light on the sleep problems attached to the limited treatment options available today. As our understanding of the pathophysiology of sleep and wake disruption heightens, novel therapeutic approaches are certain to transpire.

## Introduction

Sleep-wake disturbances are increasingly being recognized as common symptoms in neurodegenerative diseases, particularly movement disorders. Given that the quality of sleep is intrinsically associated to the quality of life, sleep disturbances inevitably induce significant morbidity. Therefore, early identification and effective treatment of sleep abnormalities is of considerable therapeutic interest, particularly for improving quality of life, delaying institutionalization and potentially reducing healthcare costs (1). Furthermore, emerging evidence has demonstrated that disturbed sleep-wake cycles may precede, influence and/or facilitate disease progression, thus possessing the potential to serve as early indicators of ongoing neurodegeneration and providing a window of opportunity to administer disease-modifying interventions early in the course of neurodegeneration.

**Figure 1:**
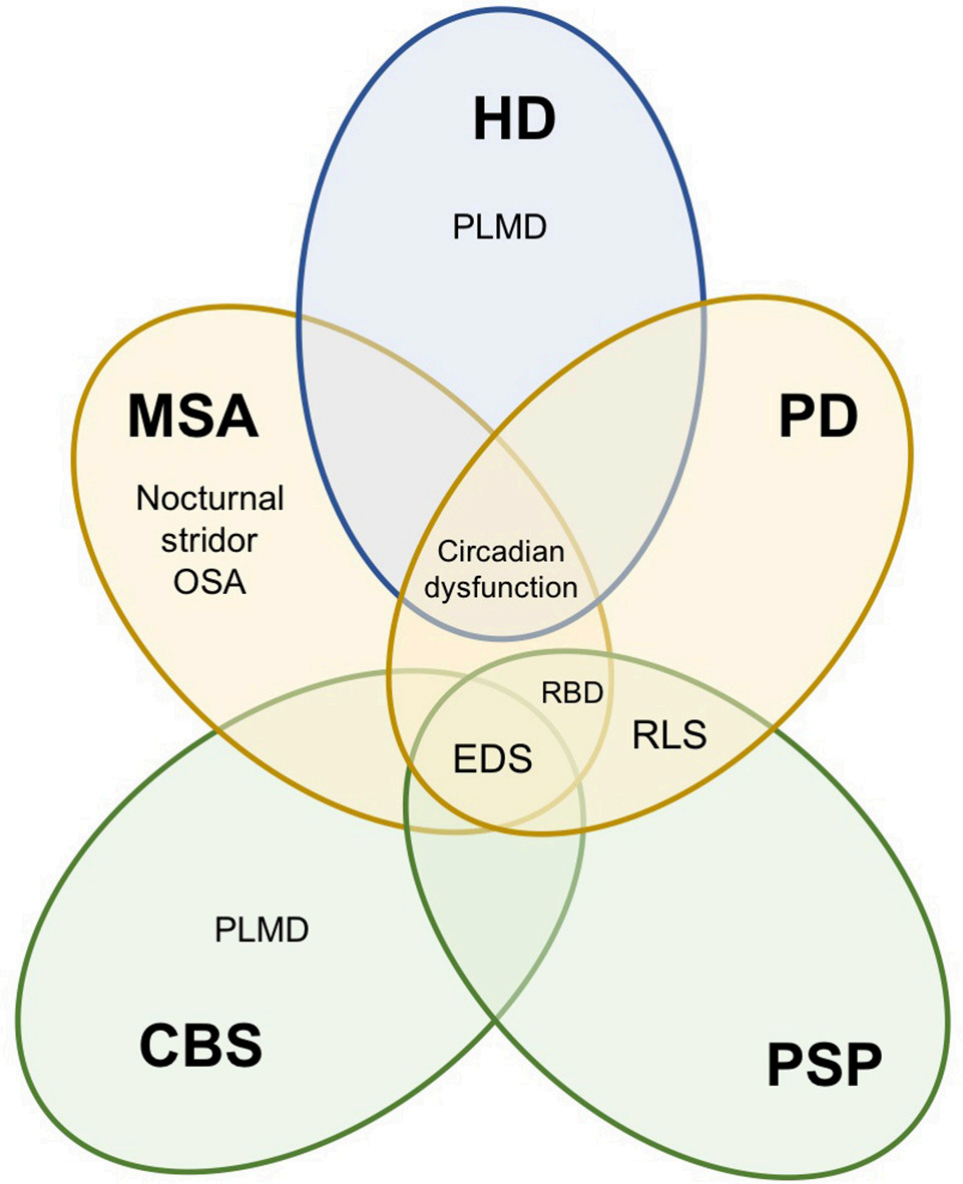
Summary of sleep disturbances found in movement disorders. Movement disorders exhibit some overlap in sleep disturbances. PSP and CBS are tauopathies (green), PD and MSA are synucleinopathies (yellow) and HD is a hereditary disorder (blue). PSP, progressive supranuclear palsy; CBS, corticobasal syndrome; PD, Parkinson's disease; MSA, multiple system atrophy; HD, Huntington's disease; EDS, excessive daytime sleepiness; RBD, REM behavior sleep disorder, RLS, restless leg syndrome; PLMD, periodic limb movement disorder; OSA, obstructive sleep apnoea.

Sleep disturbances can manifest as insomnia, sleep-disordered breathing, sleep fragmentation, restless leg syndrome (RLS), periodic limb movement disorder (PLMD), excessive daytime sleepiness (EDS) and parasomnias associated with rapid eye movements (REM) sleep. Whilst there is considerable overlap in the types of sleep disturbances reported across movement disorders, some sleep problems are either unique to, or substantially more prevalent in certain movement disorders (Figure 1). The origin of these sleep disorders is multifactorial, including neurodegeneration of central sleep regulatory structures, circadian dysfunction, impact of motor and non-motor symptoms, and adverse effects of medications (2–5).

Neuroimaging techniques, such as magnetic resonance imaging (MRI) and functional MRI (fMRI) have played a fundamental role in characterizing the structural and functional changes in patients suffering from sleep disturbances. Positron emission tomography (PET) is a powerful technique for investigating *in vivo* abnormalities in brain metabolism and receptor distribution, by enabling the measurement of radioligand distribution, which is introduced into the body on a biologically active molecule (6). By revealing the regional patterns of activation associated with specific sleep disorders, data from imaging methods, including single photon emission computed tomography (SPECT) (7–10), have complemented and extended previous findings predominantly based on electroencephalography (EEG) studies, thus improving and better characterizing the pathogenic mechanisms of major sleep disturbances.

In this article, we review the major sleep disturbances commonly experienced by patients with movement disorders, including Parkinson's disease (PD), Huntington's disease (HD) and atypical Parkinsonism, such as progressive supranuclear palsy (PSP), multiple system atrophy (MSA) and corticobasal syndrome (CBS). We discuss the role of neuroimaging in identifying neural correlates of various sleep disorders, with the aim of assessing how these studies have improved our knowledge via the insight gained into the underlying mechanisms of major sleep disturbances (Table 1).

**Table 1:** Summary of functional and structural changes associated with common sleep disturbances in movement disorders.

**Movement disorder**	**Sleep disturbance**	**Neuroimaging data**	
		**Functional changes**	**Structural changes**
Parkinson's disease	REM behavior sleep disorder	Presynaptic dopaminergic system 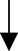 Postsynaptic dopaminergic system 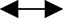 Cerebral perfusion and glucose metabolism 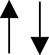 Presynaptic serotonergic system 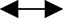 Presynaptic cholinergic system 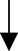 Functional connectivity 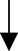	Gray matter volume 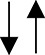 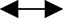 White matter integrity 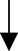 Neuromelanin density 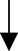
	Excessive daytime sleepiness	Presynaptic dopaminergic system 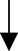 Cerebral perfusion 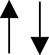 Extrastriatal dopaminergic system 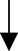 Presynaptic serotonergic system 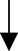 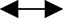 Functional connectivity 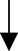	Gray matter volume 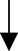 White matter integrity 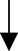 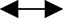
	Restless leg syndrome	Presynaptic dopaminergic system 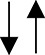 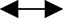 Postsynaptic dopaminergic system 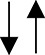 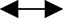 Functional connectivity 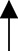	Gray matter volume 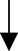 White matter integrity 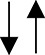 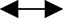 Iron concentration 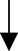
Multiple System Atrophy	REM sleep behavior disorder	Presynaptic dopaminergic system 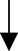 Presynaptic cholinergic system 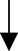	–
	Excessive daytime sleepiness	–	Gray matter volume 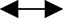
	Obstructive sleep apnea	Presynaptic cholinergic system 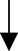	
Progressive Supranuclear Palsy	REM behavior sleep disorder	–	Gray matter volume 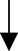 White matter integrity 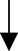
	Excessive daytime sleepiness	–	Gray matter volume 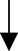
	Restless leg syndrome	–	Gray matter volume 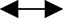
Corticobasal Syndrome^*^	Excessive daytime sleepiness	–	–
	Periodic limb movement disorder	–	–
Huntington's disease^*^	Periodic limb movement disorder	–	–
	Circadian dysfunction	–	–

* No neuroimaging studies in sleep disorders specifically.

*REM, rapid eye movement*.

## Regulatory circuits involved in sleep

**Figure 2:**
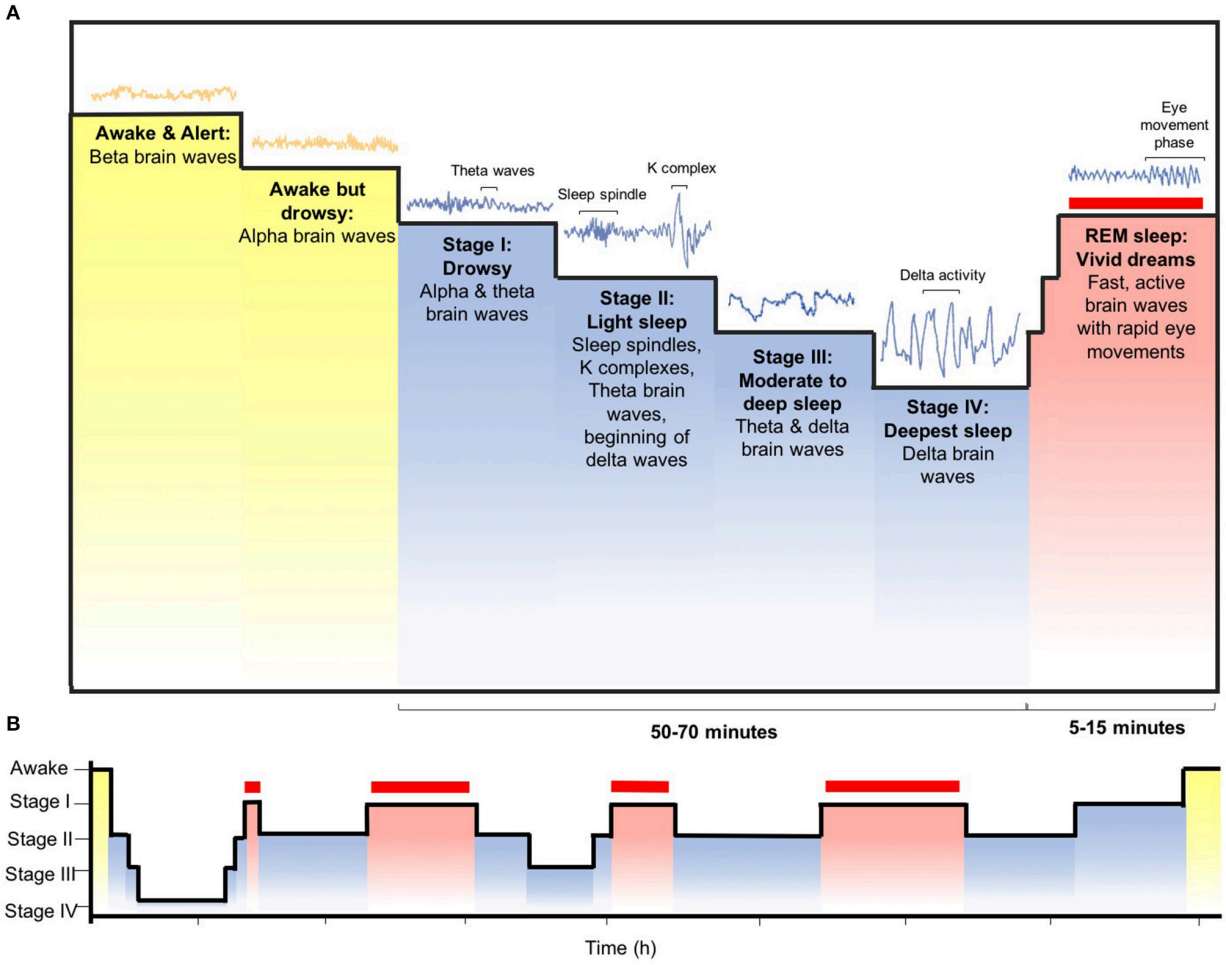
Sleep patterns and stages. **(A)** A complete sleep cycle typically takes, on average, between 90 and 110 min. There are two fundamental types of sleep: rapid eye movement (REM) sleep and non-REM sleep, which can be divided into stages I–IV. These non-REM stages correspond to the accumulating depth of sleep, as reflected by the progressive emergence of high-voltage, low-frequency brain wave activity, which dominate the deepest stages of non-REM sleep (stage III and IV, also identified as slow-wave sleep). Cardinal wave activity of stage II are sleep spindle and K-complex waveforms, who's timings are influenced by a slow oscillation (<1 Hz). REM sleep, also identified as active or paradoxical sleep, is characterized by wake-like, high-frequency, low-amplitude activity and atonia (i.e., low muscle tone). **(B)** During each of the four to five cycles that transpire each night of adult human sleep, non-REM (blue bars) and REM sleep (red bars) alternate. During the earlier proportion of the night, non-REM sleep is deeper, occupying a disproportionately large amount of time, particularly within the first cycle where the REM stage may be brief or terminated. As the night progresses, non-REM sleep becomes shallow, with more of each cycle being allocated to REM. A reliable oscillator times the sustained period of length of the non-REM and REM cycle, of which the amplitude varies according to extrinsic factors.

Sleep is indispensable for promoting adequate health and bodily function, given that it is an active physiological process required for protection and normalization of all major organs and regulatory systems (11). Sleep is a global and dynamically regulated state, primarily by two intimately integrated internal biological mechanisms: circadian rhythm and homeostasis. These control mechanisms are expressed at all levels of biological organization, from genes and intracellular mechanisms to neural networks and central neuronal systems at the organismic level. Several brain structures, both subcortical and forebrain areas, have specific functions in sleep at different organizational levels, controlling sleep-wake transitions as well as alternating between rapid eye movement (REM) and non-REM sleep (Figure 2) (12).

### The circadian system

Circadian rhythms, which can be defined as physiological and behavioral cycles with a periodicity of approximately 24 h, are orchestrated by sophisticated molecular loops. Given that the circadian system dictates the 24-h rhythmicity in biological processes such as rest-activity behavior, hormonal level, feeding and body temperature, disruption of this system can ultimately lead to negative affects imposed on alertness, sleep quality, cognitive performance, mental health, motor control and metabolism (4). These functions are often impaired in neurodegenerative diseases, with multiple brain areas, including nuclei involved in circadian and sleep regulation, being affected by neurodegenerative processes. Therefore, it is not surprising that neurodegenerative disorders often illustrate a progressive breakdown of the normal cycles of sleep and wakefulness, which not only contribute to poor quality of life and morbidity; but could also influence the disease process itself.

Molecularly, an interlocking positive-negative feedback mechanism controlling gene transcription and their protein products in individual cells of the suprachiasmatic nucleus (SCN) of the hypothalamus, which can be entrained to ambient conditions by light, is the foundation of circadian rhythmicity in mammals (12). Intrinsic circadian rhythmicity is outputted by action potentials generated by SCN cells, impinging on nuclei adjacent to the anterior hypothalamus, such as the subparaventricular nucleus (SPZ), paraventricular nucleus, dorsomedial nucleus (DMH) and the medial preoptic area, which, sequentially, convey circadian rhythmicity to structures involved in regulating physiological processes, such as the GABA-containing ventrolateral preoptic area (VPLO) and the locus coeruleus. The SCN circadian oscillator can receive feedback via melatonin, which is a sleep-related hormone secreted reliably from the pineal gland in response to polysynaptically conveyed signals from the SCN. Additionally, other neuromodulatory systems, such as acetylecholine, modulate the SCN's responsiveness to photic input from the retinohypothalamic tract (RHT). Temporal specificity is also demonstrated, given that the circadian pacemaker is responsive to specific modulatory signals exclusively at certain periods of the circadian day (12).

### Anatomy of sleep and neuroendocrine mediators

Neural circuits involved in sleep regulation can be broadly categorized under four subsystems: (1) wake-promoting; (2) non-REM sleep promoting; (3) REM sleep promoting; (4) non-REM-REM switch (13, 14). The neurodegenerative nature of movement disorders ultimately disrupts sleep regulation by interrupting the normal feedback between neuronal subsystems following accumulation of local neuropathology and concomitant changes in neurotransmitters.

The VLPO serves as a key hypothalamic structure, responsible for promoting non-REM sleep. The VLPO may initiate the onset of sleep via its reciprocal inhibition of brainstem-residing serotonergic, cholinergic and noradrenergic systems, alongside histaminergic arousal systems of the posterior hypothalamus and cholinergic systems of the basal forebrain, of which all are modulated by orexinergic arousal system of the lateral hypothalamus. Activated brain states of waking are promoted by these arousal systems, whereas the activated state of REM sleep is promoted by the cholinergic system alone (12).

Sleep-wake homeostatic information from endogenous chemical signals, such as the accumulation of adenosine during wake hours, and circadian input from the anterior hypothalamus both trigger the VLPO to initiate sleep onset. Once sleep has commenced, the alternation between non-REM and REM sleep is controlled by an ultradian oscillator in the mesopontine junction, which, in turn, is executively controlled by the interaction between aminergic REM-off and cholinergic REM-on cell groups (Figure 2). Interposed autoregulatory, inhibitory and excitatory circuits that involve serotonin, noradrenaline, acetylcholine, glutamate and GABA mediate the interaction between the two cell groups.

## Parkinson's disease

Parkinson's disease (PD) is the second most common neurodegenerative disorder, pathologically characterized by dopaminergic neuronal atrophy in the substantia nigra and widespread intracellular inclusions containing aggregates of α-synuclein (15). PD may be perceived as an admirable illustration of where efforts to comprehend impaired sleep-wake homeostasis precipitated significant advances in understanding the disease biology and progression. Conversely, sleep disorders in HD and atypical Parkinsonism, including PSP, MSA and CBS, have not been systematically studied.

### REM sleep behavior disorder

Idiopathic REM sleep behavior disorder (iRBD) is a parasomnia characterized by the lack of normal skeletal muscle atonia during REM sleep, resulting in dream-enacting behaviors often associated with violent or aggressive dreams (16). Over recent years, it is now increasingly recognized that RBD is associated with neurodegenerative disease, particularly α-synucleinopathies such as PD, MSA and dementia with Lewy Bodies (DLB) (17–19). Presumed to be a preliminary symptom of progressive neurodegeneration, RBD can precede the clinical manifestation of these α-synucleinopathies by several years, with risk estimates of up to 33% at 5 years RBD diagnosis, 76% at 10 years and 91% at 14 years (20, 21), making RBD one of the strongest clinical predictors of synucleinopathy onset (22).

Diurnal and nocturnal sleep problems are highly prevalent in PD, affecting up to 88% of the PD population, with RBD, specifically, affecting up to 50% (23, 24). Studies have demonstrated that sleep difficulties are essential predictors of poor quality of life, with most reports proposing that disturbance of sleep, lack of independence and depression are the fundamental determinants of poor quality of life (25).

Though PD is currently diagnosed when patients present with key motor symptoms including bradykinesia, rigidity, tremor or postural instability (26), the term *prodromal* has surfaced to describe the period between the onset of motor symptoms and striatal neurodegeneration, with basal ganglia degeneration suggested to commence up to 7 years prior to diagnosis (27, 28). However, prodromal symptoms such as hyposmia and RBD can emerge decades earlier in some cases, with approximately 20% of PD patients reporting RBD onset prior to motor symptomology (29). This highlights that RBD has the potential to serve as the most promising prodromal marker (30, 31). Prominently, long-term cohort studies insinuate that up to 90% of individuals who suffer from isolated RBD will go onto develop PD or atypical Parkinsonism (21, 32). However, the pathophysiology of RBD in prodromal and established PD remains unclear.

### Molecular imaging

Early imaging studies using SPECT and PET to image presynaptic dopamine transporter (DAT) and presynaptic vesicular dopamine transporters in RBD confirmed that these patients exhibit subclinical striatal dopaminergic deficit (33, 34). Consequent larger cohorts comparing RBD patients with healthy controls illustrated that 20–40% of RBD subjects exhibit abnormal nigro-striatal functioning, particularly at the putamen level (35–37).

One of the earliest studies, however, was carried out by Eisensehret and colleagues who employed SPECT tracers [^123^I]IBZM and [^123^I]IPT-SPECT, as markers of post-synaptic dopamine type-2 receptors (D_2_R) density and presynaptic DAT, respectively, to gain an insight into striatal dopaminergic integrity in five patients with iRBD compared to 14 PD patients and seven controls (34). They found a reduction in striatal [^123^I]IPT uptake in iRBD compared to controls, though no differences were found in striatal [^123^I]IBZM. Despite the small sample sizes, this study was one of the first to report a reduction in striatal dopamine transporters in idiopathic clinically manifest RBD. Further, iRBD patients exhibit a decline in striatal [^123^I]FP-CIT uptake that reflects progressive nigrostriatal dopamine depletion (38). Whilst iRBD was reported to be associated with a loss of DAT within the entire basal ganglia compared to controls at baseline, the mean reduction in striatal [^123^I]FP-CIT uptake from baseline to 3 years was 19.36% in the right putamen, 15.57% in the left putamen, 7.14% in the right caudate and 10.81% in the left caudate (38). Given the extent of caudate denervation at baseline, these findings suggest that nigro-caudate deafferentation may reflect RBD pathophysiology and putaminal deafferentation may serve as a marker of increasing severity of PD. In this direction, caudate [^123^I]FP-CIT-SPECT uptake may serve as a marker of RBD specifically and putaminal [^123^I]FP-CIT-SPECT uptake may serve as a more sensitive marker of the progression of dopaminergic dysfunction, thus both useful for studying the potential disease-modifying compounds in iRBD.

Studies have also demonstrated that nigro-putaminal functionality is impaired to a mild extent in patients with subclinical RBD (REM sleep without muscle atonia, but not RBD on polysomography), with clinically manifest idiopathic RBD patients exhibiting moderate impairment and PD patients demonstrating the most severe dopaminergic impairment (39, 40). A [^18^F]DOPA PET study scanned PD patients twice with a 5-year interval to assess the rate of disease progression and found that the disease process initially affects the posterior putamen, followed by the anterior putamen and caudate nucleus (41), whereas PD patients with iRBD displayed a more sever nigro-caudate deafferentation compared to the PD patients without RBD, whilst preserving the nigro-putaminal functionality (40), which is in line with Eisensehr and colleagues (39). These findings corroborate with the notion that nigro-caudate deafferentation may be a hallmark of RBD itself, independent of PD diagnosis. Dopaminergic imaging may, therefore, hold the potential to monitor progression throughout the prodromal phase.

Although, taken together, these results implicate the nigrostriatal dopaminergic system in the pathophysiology of RBD, it remains unclear as to what extent this system is directly involved with the increase in motor activity during REM sleep. A [^123^I]FP-CIT-SPECT study by Eisensehr observed that muscle activity during REM sleep lasting persistently longer than 0.5 s was independently associated with reduced striatal [^123^I]IPT uptake in iRBD patients (39). Although these results were not reproduced by Kim and colleagues, who found that electromyography (EMG) activities during REM sleep were not associated with striatal DAT density (36), they were partially replicated by Zoetmulder and colleagues who illustrated that EMG-activity in the mentalis muscle correlated with putaminal [^123^I]FP-CIT uptake in iRBD patients, (42). Although these findings suggest that amplified muscle activity during REM sleep is associated to nigrostriatal dopaminergic system in RBD and dopaminergic function in PD, it may not be essential for the development of RBD, thus not directly associated with the severity of RBD symptomology in PD.

An interesting study applying [^99m^Tc]-EDC SPECT with simultaneous polysomnographic (PSG) recordings demonstrated increased perfusion in the supplementary motor area during a REM sleep behavior episode (43), which was subsequently confirmed by Mayer and colleagues who reported bilateral activation of the premotor (supplementary motor) areas, the periaqueductal area, the interhemispheric cleft, the anterior lobe of the cerebellum and the dorsal and ventral pons (44). These studies implicate a common motor pathway in RBD, localizing the motor generators responsible for dream enactment behavior to include the supplementary motor area, bypassing the basal ganglia. These results corroborate with a previous study in PD patients with RBD, illustrating the “normalization” of movement during REM sleep, indicating that the motor cortex may generate movements during RBD, following the pyramidal tract bypassing the extrapyramidal system (45).

Prior observations of dopaminergic deficits in RBD have been extended by an imaging study with [^18^F]FDG PET and [^99m^Tc]-EDC SPECT, which aimed to determine whether Parkinson disease-related covariance pattern (PDRP) of cerebral glucose metabolism and perfusion, respectively, is analogous in iRBD (46). PDRP topography is characterized by elevated activity in pontine, pallidothalamic and cerebellar metabolic activity and reduced activity in parietal and premotor association regions. iRBD patients exhibited increased expression of the PDRP, with long-term clinical follow-up data highlighting that iRBD patients with network-level functional abnormalities at baseline were more likely to phenoconvert to PD/DLB (46). These results indicate network abnormalities in iRBD patients, which hold the potential to predict those who are likely to develop PD.

How does dopaminergic denervation contribute to RBD? Rye hypothesized that GABAergic output from the basal ganglia targets the glutamatergic retrorubral field and/or midbrain extrapyramidal area, which subsequently activate the ventromedial medullary zone, promoting REM atonia (47). Nigral dopamine depletion occurs transiently or persistently in pathological states such as PD. Therefore, following dopaminergic neuronal loss in the substantia nigra, we can expect heightened phasic discharge of the internal segment of the globus pallidus to excessively inhibit neurons of the midbrain extrapyramidal area, thereby permitting the expression of movements that overcome REM atonia. This is supported by the reversal of excessive nocturnal movements following excessive inhibition of the midbrain extramyramidal area by pallidotomy (48).

The variability in RBD progression to Parkinsonism may be mediated by the spread of damage in the ventral mesopontine junction to the substantia nigra. In PD, neuronal degeneration has been suggested to originate at either the dorsal or ventral portion of the brainstem, extending rostrally or caudally (49). Therefore, PD would manifest first if the lesions originate in the rostroventral midbrain, nearby the caudoventral part of the substantia nigra *pars compacta*, where the putamen-labeled afferents reside (50). RBD, on the other hand, would manifest first if the lesions begin in the caudoventral mesopontine junction (49). The variability in paths of neurodegeneration could justify why RBD either precedes PD or develops at different stages of PD.

Whilst SPECT and PET imaging studies have demonstrated a contributory role for dopaminergic dysfunction in RBD pathophysiology, pharmacological evidence has indicated that serotonergic dysfunction may also play a fundamental role in RBD development and severity. Treatment with D_2_/D_3_ receptor agonists, including pramipexole, have proven to provide little benefit in alleviating RBD-specific symptomology, possibly because post-synaptic dopaminergic integrity remains in-tact, as demonstrated by Eisensehr and colleagues, who found no difference in striatal D_2_ receptor density between iRBD and controls (34). Nevertheless, therapies predominantly targeting serotonergic functionality, such as melatonin and clonazepam, effectively treat RBD symptomology, whilst serotonin reuptake inhibitors (SSRI) are typical causative agents of medication-induced RBD (51). Furthermore, nigrostriatal dopaminergic denervation is a primary feature of PD; whereas RBD manifests in a proportion of PD patients, indicating that additional factors are likely to contribute to the underlying pathogenesis of RBD.

To investigate this notion, a combined PET study enabled authors Kotagal et al. (52) to gain an insight into the role of the cholinergic, serotoninergic and dopaminergic systems in RBD pathophysiology. In this study, 80 non-demented PD patients were enrolled and divided into two groups: PD patients positive for RBD and PD patients negative for RBD, after being assessed by Mayo Sleep Questionnaire. All subjects were scanned with three selective radioligands: [^11^C]PMP, [^11^C]DASB and [^11^C]DTBZ, which served as markers for presynaptic acetylcholinersterase (AChE), presynaptic serotonin transporter (SERT) and presynaptic vesicular monoamine transporter type 2 (VMAT2), respectively. PD patients with RBD exhibited a reduction in AChE levels within the neocortex, limbic cortex and thalamus in comparison with PD patients without RBD (52). No differences were found in striatal VMAT2 levels or SERT availability within the striatum or raphe nucleus between the two groups. Furthermore, SERT availability within the brainstem and thalamus, as measured by [^123^I]FP-CIT-SPECT, was found to not significantly differ between iRBD patients and controls (53, 54). These results, taken together, indicate that serotonergic integrity does not have an impact on RBD pathogenesis, though cholinergic dysfunction appears to play a prominent role in the development of RBD symptoms in PD.

Molecular imaging has provided us with an insight into the mechanisms underlying RBD in PD, with most studies assessing dopaminergic function and highlighting that presynaptic dopaminergic deficit is strongly associated with RBD, thus likely to play a role in its pathogenesis. Molecular imaging investigating non-dopaminergic systems in RBD is incredibly limited, thus more studies are required to assess their roles in RBD pathophysiology, particularly the cholinergic and noradrenergic systems.

### MR imaging

Multi-modal MR imaging techniques have been utilized to investigate the mechanisms underlying RBD in patients with PD, often complimenting the findings reported from molecular imaging studies. A cross-sectional resting-state fMRI study investigating functional connectivity in nigrostriatal and nigrocortical pathways reported altered connectivity in both pathways (substantia nigra, putamen and occipital regions) in RBD patients compared to PD patients and controls (55), consistent with hypotheses of dopamimergic degeneration. This was further inenforced by a combined fMRI and [^123^I]FP-CIT-SPECT study, which aimed to explore whether connectivity dysfunction within basal ganglia networks is reflected in iRBD (56). Rolinski et al. (56) revealed that iRBD patients exhibit significant connectivity dysfunction of basal ganglia network, as well as frontal lobe regions, compared to healthy controls. No significant differences in basal ganglia connectivity was identified between iRBD and PD patients, suggesting that RBD and PD patients exhibit an analogous level of decline in basal ganglia functional connectivity. However, large scale longitudinal studies are compulsory to track the progression of functional connectivity in iRBD patients who convert to PD, in order to corroborate if basal ganglia connectivity could serve as a potential marker for identifying iRBD patients at risk of converting to PD.

As well as functional alterations, RBD-specific patterns of atrophy have also been explored, in order to better understand the contribution of subcortical and cortical atrophy to alterations in the sleep-wake cycle. Voxel-based morphometry (VBM) revealed early RBD-positive PD patients, with an average disease duration of 6.5 months, had reduced cortical gray matter volume in the parietal operculum, middle occipital gyrus, insular cortex, superior temporal gyrus and hippocampus compared to RBD-negative PD patients (57). Further, RBD-positive PD patients have been reported to exhibit prominent volume loss in the anterior cingulate cortex, pontomesencephalic tegmentum, thalamus, hypothalamus, amygdala, putamen and medullary reticular formation compared to those without RBD and controls (58). There has been a relative lack of consistency in the results dictated from structural imaging studies in iRBD, with only one demonstrating a smaller volume in the pontine tegmentum (59), two others revealing a subtle structural change in this region using MRI diffusion sequences (60, 61), and one repoting no volume change in this area (62). Furthermore, previous neuroimaging studies in PD-RBD reported no significant volume changes in the pontomesencephalic tegmentum (57, 63, 64). However, it is important to note that cholinergic, glutamatergic and GABAergic neurons reside in the pontomesencephalic tegmentum, thus postulating that dysregulation of non-dopaminergic systems may contribute to RBD, whilst the volume loss in the medullary reticular formation may be implicated in the loss of muscle atonia during REM sleep. A preclinical study using transgenic mice models with impaired glycine and GABA receptor function presented with an RBD phenotype, which was rescuable using melatonin and clonazepam (65). In this direction, dysfunctional neurotransmission of glycine and GABA could play a key role in the pathogenesis of RBD, though *in vivo* studies are required to confirm this in humans.

Diffusion tensor imaging, together with T1-weighted MR imaging has enabled Ford et al. (57) to study microstructural white matter changes and atrophy, simultaneously. PD patients with RBD exhibited reduced fractional anisotropy (FA) within the inferior and superior longitudinal fasciculus, inferior fronto-occipital, longitudinal fasciculi and corticospinal tract, as well as increased mean diffusivity (MD) within the inferior longitudinal fasciculi. These results highlight widespread pathological changes in white matter microstructure, as well as reduced gray matter volume constrained to parietal and temporal lobes (57). These findings are in line with previous DTI studies in subjects with iRBD (60, 61), suggesting that microstructural impairment may contribute to RBD symptomology.

Neuromelanin-sensitive MR imaging has demonstrated that PD patients with RBD exhibit a greater loss of neuromelanin within the locus coeruleus compared to PD patients without RBD (63). Specifically, loss of neuromelanin within the locus coeruleus was linked with abnormal muscle tone during REM sleep (63). This is particularly interesting as the locus coeruleus is acknowledged to play a critical role in regulating the sleep-wake cycle via its projections to the ventral tegmental area, hypothalamus, thalamus, hippocampus and amygdala, thus substantiating the contribution of the noradrenergic system in the pathophysiology of RBD.

Evidence from SPECT and PET studies have suggested a role for dopaminergic dysfunction in RBD pathogenesis, though dysfunction of non-dopaminergic systems, including the noradrenergic and cholinergic systems, are likely to also play a role in the development of RBD in PD. Additionally, MR imaging modalities have suggested that functional connectivity and microstructural changes contribute to the development of RBD in PD, giving an insight into the neural structures which may play a key role in RBD. However, largescale longitudinal studies are indispensable to fully comprehend the mechanisms underlying RBD, thus propelling the development of targeted therapies.

### Excessive daytime sleepiness

Excessive daytime sleepiness (EDS) is one of the most common and burdensome non-motor symptoms in both early and advanced PD, substantively impacting on the patient's quality of life (66). EDS is described as an inappropriate and undesirable sleepiness during waking hours (67), which has been found to cause mild to severe cognitive impairment, with particular deficits in attention, memory and judgment (68). The prevalence of EDS has been reported to be higher in PD than in the general population, ranging from 16 to 74% (69, 70). This heterogeneity may reflect the fact that EDS has been found to rise with disease severity (71–73), as well as correlate with disease duration (74, 75), Hoehn and Yahr stage (76) and MDS Unified Parkinson's Disease Rating Scale (MDS-UPDRS) Part III (77, 76). However, the lack of correlation found between Hoehn and Yahr stage and EDS has led to the notion that sleepiness severity is not dependent on nocturnal sleep disruption, cognitive and motor impairment or anti-Parkinsonian therapy, but is actually associated with PD-specific pathology (75, 78–85).

### Molecular imaging

The pathophysiology of EDS may be a result of substantial degeneration of both the nigrostriatal dopaminergic system and extrastriatal neurons within the lower brainstem and midbrain, which pose as key regulators of the sleep-wake cycle (86). PET and SPECT molecular imaging studies have implicated both serotonergic and dopaminergic nigrostriatal dysfunction in EDS pathophysiology.

We recently carried out a [^123^I]FP-CIT SPECT study to investigate the potential implication of the presynaptic dopaminergic system in EDS. We found that PD patients with EDS generally had a worse clinical picture compared to PD without EDS, presenting with worse autonomic and cognitive function, depression and overall burden of non-motor symptom burden (87–90). Further, PD patients with EDS exhibited reduced DAT uptake within the caudate, with lower caudate DAT uptake correlating with worse EDS severity. More interestingly, abnormal caudate uptake and disease duration were found to predict the development of EDS, indicating that dopaminergic deficits in the caudate may contribute to EDS etiology (90). This is in corroboration with a study carried out by Happe and colleagues who reported a loss of [^123^I]FP-CIT uptake within the caudate, putamen and striatum, which inversely correlated with the severity of daytime sleepiness in moderate PD (H&Y stage 2) but not in early PD (H&Y stage 1), as measured by the Epworth sleepiness scale (91). Additionally, by investigating cerebral blood flow patterns in PD patients with EDS by employing [^123^I]iodoamphetamine SPECT, Matsui and colleagues reported hypoperfusion within the right caudate and the left parietal and temporal association cortex, as well as hyperperfusion within the right thalamus (92). These results suggest that cortical hypoperfusion relative to hyperperfusion within the brainstem may be related to EDS pathogenesis in PD. Interestingly, caudate functionality has been consistently demonstrated in these studies to be associated with EDS, with a reduction of caudate metabolism potentially reflecting dopamine depletion.

The caudate nucleus has recently surfaced as a common node in the regulator systems affiliated with sleep problems, given its complex role in modulating the impact of impulses between the thalamus and cortex. Neurotoxic lesion studies have revealed that bilateral striatal lesions cause a reduction in wakefulness, which is attenuated when the caudoputamen lesions include the nucleus accumbens (93). These findings emphasize that the caudate is implicated in promoting wakefulness, whereas the nucleus accumbens predominately enhances sleepiness. Therefore, hypometabolism and dopamine depletion within the caudate may cause sleepiness.

The extrastriatal dopaminergic system has also been explored *in vivo* (94, 95). A recent preliminary PET study employing [^11^C]PHNO, a radioligand selective for dopamine type-3 (D_3_) receptors, reported lower D_3_ receptor availability in the hypothalamus of 12 PD patients with EDS, which also correlated with daytime sleepiness severity (95). Although this study suggests that D_3_ receptor availability may be associated with EDS, it does not provide a principal cause for this decline. This led the authors to suggest that reduced monoamine transmission within important nuclei implicated in sleep control are accountable for adaptive changes in D_3_ receptor availability. A possible alternative explanation is that there is direct neuronal degeneration of dopamine receptor-expressing neurons within the hypothalamic dopaminergic networks (95).

Given that preclinical data implicates the brainstem raphe nuclei in the ascending arousal system, which promotes wakefulness and prevents EDS (96), the serotonergic system has also been investigated *in vivo*. A combined PET study utilizing [^11^C]DASB and [^18^F]DOPA, as markers of SERT and monoaminergic terminals function, respectively, revealed that PD subjects with EDS exhibited a reduction in SERT availability within the rostral raphe, locus coeruleus, thalamus and hypothalamus, as well as reduced [^18^F]DOPA uptake within the locus coeruleus, rostral raphe and ventral tegmental area (97). Since the authors controlled for depression and fatigue, it can be suggested that monoaminergic dysfunction may contribute to the mechanisms underlying EDS. However, these findings have not been replicated (54).

Molecular imaging has consistently demonstrated the caudate to be associated with EDS, as shown by a negative correlation between its metabolic activity and dopaminergic function and the severity of EDS. However, there is a real lack of non-dopaminergic exploration using molecular imaging techniques, which is critical to further expand our understading of daytime sleepiness, especially because wake-promoting neurotransmitters include norepinephrine, acetylcholine, hypocrein, histamine and hypocretin, which may be dysregulated in PD.

### MR imaging

At the structural level, whole-brain studies revealed widespread reductions of gray matter volume in the frontal, occipital, temporal and limbic lobes, as well as the right parahippocampus and nucleus basalis of Meynert in PD with EDS compared to PD without EDS (98, 99). Whole-brain white matter analysis of DTI data have revealed that PD patients with EDS exhibit elevated levels of white matter axonal damage, indexed by reduced fractional anisotropy within the fornix compared to those without EDS, which also correlated inversely with daytime sleepiness severity (100). Furthermore, Chondrogiorgi and colleagues revealed that PD patients with EDS exhibited a reduction of white matter integrity [as demonstrated by an increase in axial diffusivity (AD)] within the projection, association and brainstem fibers compared to PD patients without EDS (99), which corroborated with reports made by Gama et al. (101) and Kato et al. (98), who found widespread cortical and subcortical atrophy within frontal, temporal, limbic and insular regions, as well as the middle cerebellar preduncles. Although a larger cohort study is required to confirm these findings, these findings are suggestive of fornix fibers degeneration, which connects the hypothalamus and hippocampus, playing a potential role in EDS development. Hypothalamic dysfunction could lead to microstructural changes in the fornix, thus together contributing to the development of sleepiness in PD. Additionally, longitudinal studies are a necessity to gain an insight into the long-term role of structural and functional alterations in EDS pathophysiology in PD.

At the functional level, increased EDS was inversely associated with decreased functional connectivity within the thalamocortical and default mode networks, potentially reflecting the disengagement of sensory and motor processing from the stream of consciousness (102, 103). Additional rs-fMRI studies using whole-brain analysis identified regional homogeneity within the inferior gyrus and cerebellum, which inversely correlated with ESS score, and increased regional homogeneity in the paracentral lobule, which positively correlated with ESS scores (104). Subsequent functional connectivity analysis of these regions demonstrated a reduction in functional connectivity in the frontal, temporal, insula and limbic lobes, as well as the cerebellum, in EDS-positive PD patients compared to EDS-negative patients (104). The frontal, temporal and insula regions have been reported to play a role in the development of EDS, given the marked atrophy within the gray matter of these regions (92, 98).

Despite some negative findings, such as no significant EDS-related changes in FA (99), most studies observed a reduction in structural and/or functional features, including white matter integrity, white matter axonal damage, brain volume and functional connectivity in key neural structures involved in regulating alertness and sleepiness. However, further multimodal studies are required to gain a deeper understanding of the way in which structural changes are associated with EDS, particularly by carrying out longitudinal studies.

## Restless leg syndrome

Idiopathic restless leg syndrome (iRLS), also known as Willis-Ekbom disease (WED), is a common neurological disorder characterized by strong feelings of restlessness and troublesome paraesthesia-like sensations in the lower legs, particularly at rest. Contrariwise, symptomology usually improves or disappears once the subject starts physical activity. This condition affects sleep and health (105–107), as well as quality of life, which has been reported to be analogous or worse than those suffering from congestive heart failure, osteoarthritis, stroke or depression (106, 108–116).

Most iRLS patients display some initial clinical benefit when administered dopaminergic drugs, including dopamine agonists and levodopa. Large-scale clinical trials encompassing diverse patient populations reported good clinical response to dopamine agonist in approximately 60–75% of participants (117). Therefore, in general clinical practice, a failure to respond to dopaminergic treatment should flag concern regarding the accuracy of diagnosis, but does not automatically exclude a diagnosis of iRLS.

### Molecular imaging

SPECT and PET molecular imaging techniques have been employed to investigate the role of dopaminergic dysfunction in the pathophysiology of iRLS, by imaging post-synaptic D_2_ receptor binding, and presynaptic DAT (118). However, few studies have aimed to characterize the underlying mechanisms of RLS in PD cohorts specifically. Generally, brain imaging studies evaluating dopaminergic function in iRLS patients have yielded inconclusive results though some have provided evidence for the pathogenic role of the dopaminergic dysfunction, which is in line with the effectiveness of dopaminergic therapies (119).

Striatal DAT density has been found to be compromised in iRLS patients, as demonstrated by two molecular studies employing [^99M^Tc]TRODAT-1 SPECT (120) and [^11^C]methylphenidate PET (121). This presynaptic dopaminergic deficit is in line with studies employing [^18^F]DOPA, where iRLS patients exhibited reduced [^18^F]DOPA uptake in the putamen and caudate compared to controls, highlighting the potential role that dopaminergic dysfunction plays in RLS pathophysiology (122). However, a 4-year longitudinal [^123^I]-FP-CIT SPECT study in 88 *de novo* PD patients revealed that elevated DAT availability within the caudate and putamen was associated with the presence of RLS at baseline and follow-up, compared to those without RLS (123), suggesting that PD patients with RLS may have a comparatively preserved presynaptic dopaminergic pathways.

Striatal and extrastriatal D_2_ receptor availability in *de novo* RLS patients has been investigated using [^11^C]raclopride and [^11^C]FLB-457, respectively, with RLS participants illustrating increased availability of D_2_ receptor within the thalamus and cingulate cortex (124), but a reduction of D_2_ receptor binding within the caudate and putamen (125). The elevation of D_2_ receptor availability in RLS could be a result of the upregulation of D_2_ receptor in response to low levels of endogenous dopamine. Striatal and extrastriatal dopaminergic dysfunction and alterations in somatosensory processing may contribute to the mechanisms underlying RLS. Michaud et al. (126) employed [^123^I]-β-CIT and [^123^]IBZM to assess pre- and post-synaptic dopaminergic impairment, respectively. Reduced striatal D_2_ receptor binding was observed in RLS patients compared to healthy controls, though no differences were identified in presynaptic DAT binding (126). Given that presynaptic DAT uptake was within the normal range, reduced D_2_ receptor availability is likely to reflect downregulation of D_2_ receptor due to elevated synaptic dopamine levels, supporting the role of post-synaptic dopaminergic dysfunction in the pathophysiology of RLS.

Although there is good evidence supporting the notion that dopaminergic dysfunction contributes to RLS manifestation, some molecular imaging studies have revealed conflicting results. A [^123^I]-β-CIT and [^123^]IBZM SPECT study reported a reduction in putaminal and caudate DAT uptake, and no difference in striatal D_2_ receptor density, in *de novo* iRLS patients (*n* = 13) compared to controls (*n* = 12) (127). Conversely, other studies have demonstrated that no difference resides in dopaminergic activity between RLS patients and controls, with no striatal or frontal D_2_ receptor differences between RLS patients, early PD and healthy controls (128). These results, taken together, suggest that the underlying mechanisms of RLS are likely to involve non-dopaminergic systems, with the underlying mechanisms likely to be divergent from PD where presynaptic dopaminergic neuronal integrity is largely affected. In a study that examined real-time DAT binding potentials, RLS patients exhibited a reduction of striatal DAT binding in both day and night scans, suggesting that membrane-bound striatal DAT, but not total cellular DAT is depleted in RLS (121).

There are no studies, to date, exploring the non-dopaminergic basis of RLS in PD, which is required to enhance our understanding of this disorder, especially given the conflicting results in dopaminergic integrity between studies.

### MR imaging

Studies exploring structural changes in RLS are very limited. The first study to report morphologic changes in RLS was published earlier this year, who found that RLS patients had a 7.5% decrease in cortical thickness in the bilateral post-central gyrus. Further, there was a decline in the corpus callosum posterior midbody, suggesting an alteration in white matter properties in the somatosensory pathway (129). Other studies exploring white matter microstructural changes have revealed altered fractional anisotropy in temporal regions, internal capsule cerebellum and pons in iRLS patients compared to controls, with no changes in gray matter (130). These results indicate that microstructural alterations in white matter, but not gray matter atrophy, may contribute to the development of iRLS. Nevertheless, Rizzo and colleagues reported no microstructural changes in iRLS subjects (131), thus arguing against structural or microstructural abnormalities having an association with iRLS. A functional neuroimaging study by Bucher and colleagues reported that iRLS patients exhibit activation in the cerebellum and thalamus (132), which is supported by structural cerebral and thalamic findings reported by Etgen et al. (133).

Iron deficiency has also been repeatedly shown to be associated with RLS, especially given that CSF ferritin levels are low and CSF transferrin levels are high in RLS patients (134). However, total iron and ferritin levels in the substantia nigra region are elevated in PD, resulting in oxidative stress and potentially leading to dopamine depletion (135). In idiopathic RLS, central nervous system iron storage may be impaired, though systemic iron may be normal (136). Utilizing MRI techniques, regional brain iron concentration has been assessed in iRLS patients, which reported decreased iron in the midbrain, which has been found to correlate with the severity of RLS (137). In a recent study with a 7-T MRI scanner, iRLS patients exhibited a reduction in iron levels, as measured by quantitative magnetic susceptibility mapping (SWI), within the thalamus and dentate nucleus, but not in the substantia nigra, when compared to controls (138). The mechanism by which iron deficiency leads to dopaminergic dysfunction remains uncertain. Iron has a complex effect on dopaminergic function, serving as a cofactor for tyrosine hydroxylase and is integral to D_2_ receptor function (139, 140).

Although PD and RLS are both related to central dopaminergic dysfunction, the evidence of an association between these disorders is limited to a few studies that report inconsistent results. Long-term prosective controlled studies are warranted to not only assess the pathophysiology of RLS in PD, but also the link between PD and RLS as clinical association studies differ widely. Currently, there are not functional MRI studies rhat have focused on patients with PD-RLS/Functional and molecular imaging studies of PD vs. PD-RLS are necessary to better understand the mechanisms involved in these disorders.

## Multiple system atrophy

MSA is an adult-onset, fatal neurodegenerative disorder characterized by a combination of autonomic dysfunction, Parkinsonian features and cerebellar and pyramidal features (141). The neuropathological hallmark of MSA is α-synuclein-immunoreactive inclusions that predominantly affect oligodendrocytes (142). The underlying neuropathology may contribute to the sleep disorders observed in this disease, with sleep dysfunction acknowledged to be an associated comorbidity. Some sleep disorders such as RBD and vocalization appear to have a higher prevalence in MSA than in PD, despite the same disease duration, and are typically associated with more severe motor symptoms, depression, longer disease duration and longer duration of levodopa treatment (143).

### REM sleep behavior disorder

MSA patients have reduced REM and slow-wave sleep, with a recent multicenter study revealing that polysomnography-confirmed RBD is present in up to 88% of patients with MSA. RBD has also been shown to precede the onset of MSA by more than 1 year in 44% of patients (144).

Given that both acetylcholine- and monoamine- containing neurons of the brainstem have been implicated in REM sleep (47), Gilman and colleagues employed [^11^C]DTBZ PET and [^123^I]IBVM SPECT, markers of striatal monoaminergic presynaptic terminals and cholinergic presynaptic terminals, respectively, to explore the neurochemical basis of RBD in 13 subjects with probable MSA (145). The MSA subjects exhibited a reduction in mean striatal [^11^C]DTBZ uptake, as well as a reduction in [^123^I]IBVM uptake within the thalamus (145). These results reflect the degeneration of dopaminergic neurons in the substantia nigra, as well as degeneration of cholinergic neurons in the pedunculopontine tegmental and laterodorsal tegmental nuclei. However, given the lack of correlation found between thalamic [^123^I]IBVM uptake and severity of REM atonia loss, it can be assumed that degenerative changes in the pedunculopontine tengmental and laterodorsal tegmental nuclei do not contribute to RBD. Striatal [^11^C]DTBZ binding, however, was inversely correlated with severity of REM atonia loss, which led the authors to conclude that loss of nigrostriatal dopaminergic projections may contribute to RBD in MSA.

### Excessive daytime sleepiness

The prevalence and nature of EDS in MSA has not been systematically investigated. However, the few studies which have evaluated sleepiness have reported that 50% of MSA patients suffer from EDS (143), though Multiple Sleep Latency Tests in this subset of patients have revealed normal or mildly reduced sleep latencies (70, 146). In a multicenter survey, Epworth sleepiness scale scores from patients with MSA were comparable to PD, though higher compared to controls, with EDS present in 28% of MSA subjects (147). In contrast to PD, EDS did not correlate with dopaminergic treatment, and disease severity weakly correlated. However, sleep-disordered breathing and sleep efficiency predicted EDS in MSA (147).

One of the potential neuroanatomic substrates for EDS in MSA includes the hypocretin neuronal network within the lateral hypothalamus, which is acknowledged to play a fundamental role in promoting alertness. In an autopsy study of 7 MSA patients, the number of hypocretin neurons were significantly reduced compared to controls (148), though other studies have demonstrated that MSA patients have normal hypocretin levels in the cerebrospinal fluid (CSF) (146, 149). These findings suggest that substantial neurodegeneration is required in order for a measurable difference in CSF hypocretin levels to be detectable. Alternatively, EDS observed in MSA may be related to a loss of cholinergic alerting neurons from the brainstem, as Schmeichel and colleagues have reported that MSA subjects have a significant loss of cholinergic neurons within the laterodorsal tegmental and pedunculopontine tegmental nuclei within the pons (150). However, *in vivo* molecular imaging studies are required to confirm this notion in MSA subjects.

In an interesting cross-sectional MRI study including 16 PD cases, 13 MSA and 14 PSP, alongside 12 healthy controls, Gama and colleagues sought to evaluate and compare the three groups regarding EDS and its association to brain MRI morphometry. MSA subjects with EDS did not exhibit any EDS-specific brain atrophy, though PD subjects with EDS presented with more atrophy of the medial cerebellar peduncle compared to PD patients without EDS and PSP subjects exhibited atrophy within the midbrain compared to controls (101). The authors concluded that widespread neurodegeneration of brainstem sleep structures were related to sleep abnormalities in these subjects.

### Sleep disordered breathing

Various types of sleep disordered breathing can arise in MSA. Nocturnal stridor and obstructive sleep apnea (OSA) occur more frequently than central sleep apnea, ranging from 40 to 42% for nocturnal stridor in MSA cases (151, 152) and 15–37% for OSA, with both carrying the risk off sudden death (144, 152). Laryngeal dysfunction and narrowing of the upper airway at the level of the vocal cords, laryngeal inlet, base of the tongue and soft palate has been reported in early descriptions of MSA (153, 154). Other factors, including rigidity, hypokinesia, dystonia, rigidity and paralysis of the upper airway muscles may predispose to OSA or nocturnal stridor in MSA (155).

Gilman and colleagues used [^123^I]IBVM SPECT to measure thalamic cholinergic terminal density and [^11^C]DTBZ to measure striatal monoaminergic terminal density to investigate the neuropshyiologic basis of OSA in MSA (156). The authors reported that thalamic [^123^I]IBVM uptake inversely correlated with OSA severity, with no correlations found between [^11^C]DTBZ and OSA severity. Given the limited number of cholinergic neuronal cell bodies in the thalamus (157), thalamic [^123^I]IBVM binding may serve as a surrogate marker for the density of cholinergic neurons in the pontine pedunculopontine tegmental and laterodorsal tegmental nuclei. Therefore, projections from pedunculopontine tegmental and laterodorsal tegmental nuclei to the medulla and pons may be more applicable in the pathophysiology of OSA, especially given that OSA mirrors collapse of the upper airway musculature, which receives cholinergic innervation from motor neurons of the lower brainstem.

To the best of our knowledge, there are no other PET or MR studies exploring the neurochemical or neuroanatomical correlates of sleep disorders in MSA. Further studies, both structural and functional neuroimaging, are required to enhance our understanding of these sleep disturbances in MSA.

## Progressive supranuclear palsy

PSP belongs to the family of tauopathies, characterized by abnormalities in the hyperphosphorylation and aggregation of the microtubule-associated protein, tau, resulting in degeneration of cortical and subcortical brain structures, particularly within the midbrain (158). A PSP patient typically presents with postural instability, supranuclear ophthalmoplegia, Parkinsonism and pseudobulbar palsy (159).

Although more commonly reported in synucleinopathies, RBD has also been observed in PSP. In a relatively small study of 15 PSP patients, 15 PD patients and 15 age-matched controls, REM sleep without atonia and RBD were similarly present amongst the two cohorts (33 vs. 28%) (160), which was confirmed by Diederich et al. (161). In fact, a polysomnographically recorded study demonstrated that RBD and REM sleep without atonia are common also in PSP, and that sleep was more severely impaired in PSP than in PD (162).

Given the lack of neuroimaging studies assessing sleep disturbances in PSP, in this section, we discuss the findings from neuroimaging studies which may have relevance to sleep regulation.

PSP is pathologically characterized by abnormal accumulation of tau protein, with prominent atrophy in the midbrain (163). Most PSP patients also exhibit neurodegeneration of cholinergic neurons at the level of the pedunculopontine tegmentum, which is acknowledged to play a fundamental role in the regulation of REM sleep (164). Atrophy of the brainstem have been reflected in MR morphometry studies, which have described a reduction in the antero-posterior diameter of the midbrain and smaller ratio between midbrain area and pons (165–167).

Several subcortical structures degenerate over the course of PSP, as demonstrated by Saini et al. (168) and Whitwell et al. (169), who reported non-specific atrophy of the thalamus. However, after attempting to delineate the thalamus, Padovani and colleagues revealed that the pulvinar, dorsomedial and anterior nuclei of the thalamus was atrophic in PSP (170, 171). This was in corroboration with a previous study carried out by Price and colleagues who reported reduced gray matter in the thalamus and hypothalamus in PSP compared to controls (172). Studies have also reported prominent white matter atrophy within the midbrain and pons (169, 173, 174), with a loss of white matter volumes in the brainstem and subcortical structures of PSP patients. Whitwell and colleagues reported reduced fractional anisotropy within the pons and increased mean diffusivity within the thalamus of PSP patients (*n* = 18), with the midbrain exhibiting a decrease in fractional anisotropy and increase in mean diffusivity (175).

In an MRI study carried out by Lehericy et al. (176), 10 PSP subjects had marked gray matter atrophy in the medial thalamus and anterodorsal midbrain/hypothalamic area compared to controls. Reduced white matter volumes were evident in the basal forebrain/inferior globus pallidus/subthalamic area, midbrain and pons, amongst other cortical and subcortical regions. Further, DTI analysis revealed that PSP subjects exhibited lower fractional anisotropy in the white matter of the thalamus, midbrain, cerebellum and cortical regions (176).

The brainstem, thalamus and hypothalamus play essential roles in regulating sleep, with the brainstem and the hypothalamus centrally regulating the pace for sleep-related activity throughout the brain and the thalamus being targeted by both central and decentral regulators to induce global sleep-related oscillatory activity (177). The pons, in particular, is important for REM sleep, with several pontine structures contributing to the generation of each specific polygraphic event that characterizes REM sleep. The pontine tegmentum also encompasses the region where cholinergic activation can trigger behavioral and bioelectric signs of REM sleep (178), with the pedunculopontine tegmental nuclei, in particular, thought to be critical for generating REM sleep, and its connections to the reticular nuclei of the thalamus thought to be important for generating spindles (179). In this direction, degeneration and pathological changes within these regions could underlie RBD within PSP subjects, though neuroimaging studies are required to confirm whether an association is present in PSP.

## Corticobasal syndrome

CBS also belongs to the family of taupathies, though much less common than other atypical Parkinsonian disorders. CBS patients characteristically present with myoclonus, dystonia, progressive asymmetric levodopa-resistant Parkinsonism, and further cortical signs, such as alien limb phenomena, apraxia and cortical sensory loss (180). The neuropathological hallmark of CBS is unusual accumulation of hyperphosphorylated 4-repeat (4R) tau in the form of neurofibrillary tangles, coiled bodies and neuropil threads together with astrocytic plaques.

The literature on CBS is primarily limited to case reports, likely due to the rarity of CBS. Two case reports of RBD have been reported (181, 182), though in a larger case series of CBS patients, only 1 out of 11 patients had RBD (183). A descriptive study including five CBS patients reported that none had RBD, two had sleep disordered breathing, four had periodic limb movements during sleep and all five patients had insomnia (184).

CSF hypocretin levels have been evaluated in CBS and found to be significantly lower than in patients with PD. The sample size was quite small (*n* = 7) and the majority of the effect may have been primarily due to one outlier (185). Further, pathological studies have demonstrated that CBS patients have a significant loss of choline acetyltransferase (ChAT)-positive neurons within the nucleus basalis of Meynert compared to PSP (186).

There have been no studies, to-date, exploring the neuropathological basis of sleep disturbances in CBS using neuroimaging techniques. A data-driven meta-analysis identifying and comparing the neural correlates of CBS for atrophy measurements included 200 CBS patients and 318 controls (187). This study identified four brain regions with significant atrophy: (1) the bilateral anterior thalamus; (2) the posterior midcingulate cortex, bilateral posterior frontomedian cortex and the premotor area/supplementary motor area; (3) the posterior superior frontal sulcus and precentral gyrus/middle frontal gyrus; (4) the left posterior superior frontal sulcus and middle frontal gyrus. This is particularly interesting as thalamic neurons provide state-dependent gating of sensory information through their ability to create differential patterns of electrogenic activity during wakefulness and sleep. It has been argued that both central and decentral regulators target the thalamus, inducing global sleep-related oscillatory activity (177).

Striatal DAT uptake in patients with CBS is characterized by large variability, with [^123^I]FP-CIT found to be normal bilaterally in four CBS patients and only unilaterally reduced in the other four cases (188). These results highlight the hemispheric asymmetry in the putamen and caudate [^123^I]FP-CIT uptake, thus reflects the highly asymmetrical involvement and substantia nigra *pars compacta* neuronal loss implicating both ventral and dorsal tiers in CBS (189, 190), as opposed to PD, where cell loss is typically confined to the substantia *nigra pars compacta* ventral tiers. Given the association found between striatal dopamine depletion and sleep disturbances in PD, it could be assumed that a similar mechanism underlies sleep disorders in CBS. However, this needs to be investigated in MSA exhibiting specific sleep disorders in well-designed, controlled studies.

## Huntington's disease

Huntington's disease (HD) is a hereditary and fatal neurodegenerative disease, characterized by chorea, psychiatric symptoms and cognitive dysfunction, which manifest as a result of an expanded trinucleotide CAG sequence in the huntingtin gene (HTT) on chromosome 4 (191, 192). HD-specific pathology is defined by aggregations of intranuclear inclusions of mutated huntingtin in the brain. These aggregates have been reported to interact and impair the function of several transcription factors, ultimately inducing a loss of striatal GABAergic medium spiny neurons (MSNs), as well as cortical MSNs (193, 194). There is growing awareness that, alongside the psychiatric and cognitive syptoms, sleep and circadian abnormalities are also present. However, it remains unclear if they are directly caused by HD gene-related pathology or are merely a consequence of having a neurodegenerative disease.

The changes seen in HD appear to have very little in common with sleep disturbances in the neurological disorders mentioned above. For example, the incidence of RBD in the early stages of HD are reported to be fairly low, which is consistent with the fact that although dopaminergic pathology is evident in HD, it does not dominate at early stages (195). At subsequent stages of HD when dopamine-mediated function has deteriorated, the degeneration of this pathway is only part of a much wider picture. Other parasomnias that are associated with neurodegenerative disease, such as RLS, is not significantly increased in the HD population, though periodic limb movement disorder (PLMD) appears to be more frequent in this population compared to controls. It is important to note, however, that it is difficult to distinguish between PLMD and chorea—which is part of their movement disorder. Although sleep disorder studies in HD are rare or non-existent, we could gain critical insight into the potential sleep comorbidity in HD by investigating the molecular, structural and functional basis of sleep-related circuitry in HD. Here, we discuss the neuronal circuitry known to be implicated in sleep and affected in HD, as demonstrated by neuroimaging techniques.

HD pathology is characterized by atrophy and degeneration of the neostriatum and cortex that eventually comprises of the whole brain including subcortical structures (85, 196). Although sleep disturbances were originally reported to be associated with the degree of atrophy within the caudate (197), more recent findings revealed this correlation to be weak (198).

### Molecular imaging

The hypothalamus is critical for the regulation of sleep and metabolism, as well as playing a key role in the regulation of automatic functions such as heartbeat and breathing. A [^11^C]PK11195 and [^11^C]raclopride PET study with the aim of assessing *in vivo* D_2_ receptor integrity and microglial activation in premanifest (*n* = 10) and manifest (*n* = 9) HD gene carriers reported a significant decrease in hypothalamic [^11^C]raclopride uptake in both premanifest and manifest HD gene carriers, as well as a significant increases in mean hypothalamic [^11^C]PK11195 uptake (199). This study was the first to demonstrate a significant loss of D_2_ receptors, as well as increased microglial activation within the hypothalamus of HD (199). This is in corroboration with a multimodal imaging study which used MRI, [^11^C]PK11195 and [^11^C]raclopride PET to investigate volumetric differences, as well as microglial activation and D_2_/D_3_ receptor binding in premanifest and symptomatic HD gene carriers (200). The authors found increased microglial activation within the hypothalamus, amongst other regions, as well as a reduction in hypothalamic D_2_/D_3_ receptor binding in premanifest HD gene carriers. This is particularly interesting as, even though sleep was not specifically investigated in this cohort, the hypothalamus is known to play a critical role in regulation sleep, emotion and appetite (201–203).

### MR imaging

Studies employing voxel-based morphometry analyses have reported a reduction in voxel signal intensity within the hypothalamus in early symptomatic stages of the disease (204, 205). Soneson and colleagues have revealed, using both VBM and logistic regression analyses, that hypothalamic changes are visible from up to 15 years before the predicted onset of motor symptoms (206). Hypothalamic dysfunction in HD patients would not only have an impact on circadian rhythm and daytime sleepiness, but would affect additional relevant functions that have an influence on sleep. Although evidence from MRI studies have suggested that neurodegeneration in the hypothalamus presents very early in HD, the function or structure of the suprachiasmatic nuclei, which resides in the anterior, ventral region of the hypothalamus, has not been rigorously examined in post-mortem brain. However, significant loss and atrophy of orexin/hypocrein neurons have been identified in HD (207), alongside the degeneration of the thalamus (208–210), which is crucial for the generation of sleep and wakefulness. However, it has been poorly studied, with little known about if or how thalamic pathology impacts on sleep in HD.

Given the lack of neuroimaging studies specifically exploring the pathology underlying changes in sleep and circadian activity in HD, the pathophysiology of sleep comorbidities in HD remains unclear. There is, therefore a dire need for studies investigating HD-specific sleep issues to enhance our understanding the cause of these symptoms in HD, thus help us understand how they can be treated.

## Conclusion and future directions

Sleep is a multifaceted phenomenon, regulated by an intricate interplay between several neurotransmitter systems. Although efforts have been made to delineate structural and functional correlates of sleep disturbances, there is a need for standardization across studies, especially given the discord between various studies. Furthermore, multimodal neuroimaging studies hold the key to further understand the simultaneous disruption of various systems, which may all, in some way, play a role in sleep disturbances.

Overall, neuroimaging techniques are unparalleled tools for investigating the mechanisms underlying sleep impairments in movement disorders. These invaluable techniques have provided some insights into the pathophysiology of sleep disorders, unraveling the involvement of both dopaminergic and non-dopaminergic systems. Nonetheless, the visualization of functional and neuroanatomical hallmarks of these sleep disorders remains an active and challenging area. There is a great need of imaging studies specifically seeking to understand neuropathological substrates of sleep disorders in atypical Parkinsonism and HD, which could potentially serve as novel targets for pharmacotherapy.

## Author contributions

TY gave input into the article design; wrote the first draft; prepared and revised the manuscript and generated the figures. GP, HW revised the manuscript for intellectual content. MP revised the manuscript for intellectual content and gave final approval of the manuscript.

### Conflict of interest statement

The authors declare that the research was conducted in the absence of any commercial or financial relationships that could be construed as a potential conflict of interest.
